# Supplementary material for: Spatial Variation in Stream-Water Bacterial Communities Across Sites Differing in Wild Chinese Giant Salamander Detection Frequency
Source: Biology (Basel). 2026 Sep 18;15(18):1647. doi: 10.3390/biology15181647 (PMC13604818; doi:10.3390/biology15181647)
Supplement: Supplementary file 1 [file biology-15-01647-s001.zip › Data Related to “Spatial Variation in Stream-Water Bacterial Communities across Sites Differing in Wild Chinese Giant Salamander Detection Frequency”/Microbial Data/result/08.FunctionPrediction/picrust2/pca/sample/KO/level1/PCA_circleLable.pdf]

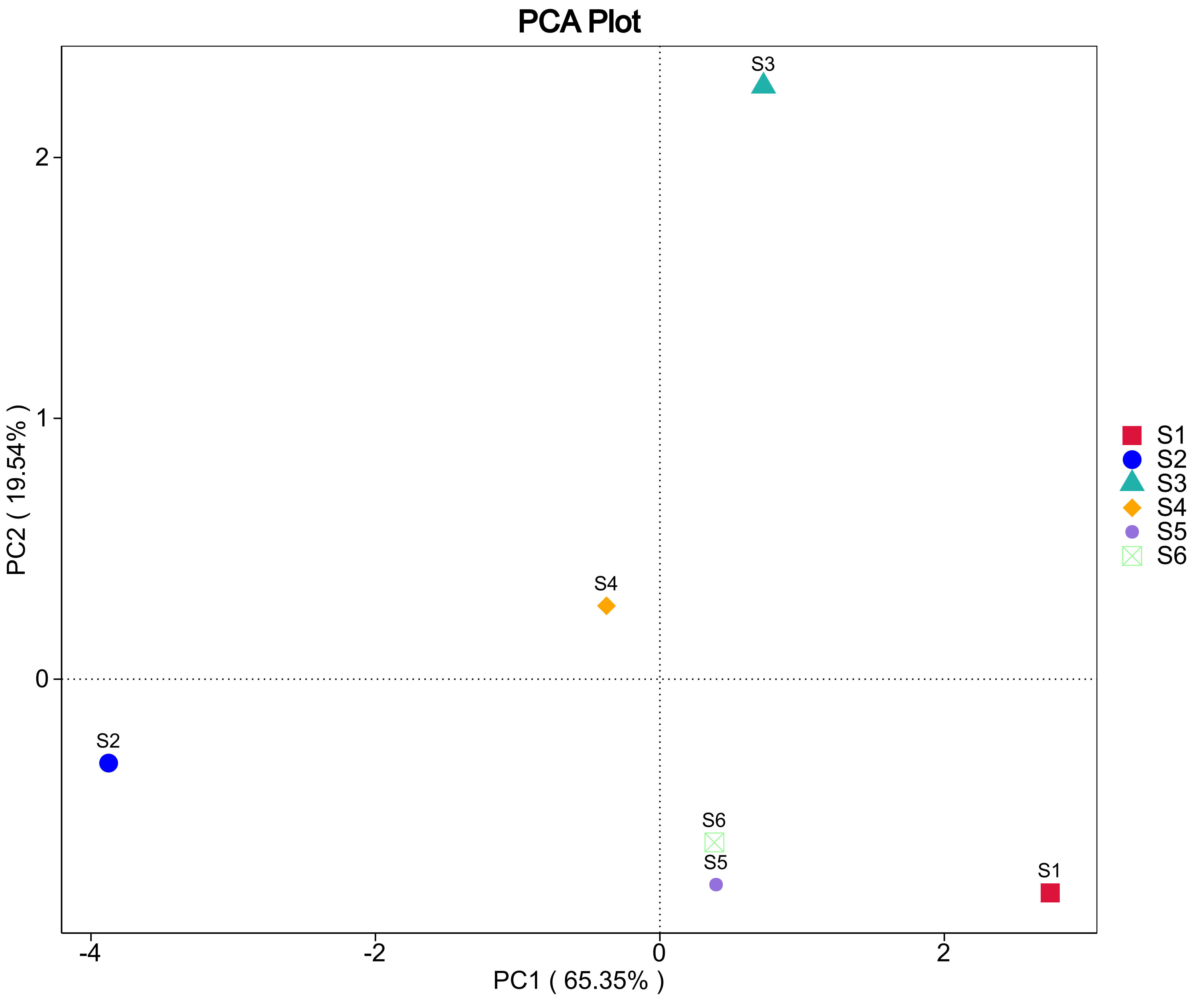
PCA Plot
S3
S4
S2
S6
S5
S1
2
1
0
-4
-2
0
2
PC1 (65.35%)
PC2 (19.54%)
S1
S2
S3
S4
S5
S6
